# The chloroplast genomes of four *Bupleurum* (Apiaceae) species endemic to Southwestern China, a diversity center of the genus, as well as their evolutionary implications and phylogenetic inferences

**DOI:** 10.1186/s12864-021-08008-z

**Published:** 2021-10-02

**Authors:** Rong Huang, Xuena Xie, Aimin Chen, Fang Li, Enwei Tian, Zhi Chao

**Affiliations:** 1 Department of Pharmacy, Zhujiang Hospital, Southern Medical University, Guangzhou, 510282 China; 2 Faculty of Medicinal Plants and Pharmacognosy, School of Traditional Chinese Medicine, Southern Medical University, Guangzhou, 510515 China; 3 Guangdong Provincial Key Laboratory of Chinese Medicine Pharmaceutics, Guangzhou, 510515 China

**Keywords:** *Bupleurum*, Chloroplast genome, Comparative analysis, Phylogenetic analyses

## Abstract

**Background:**

As one of the largest genera in Apiaceae, *Bupleurum* L. is well known for its high medicinal value. The genus has frequently attracted the attention of evolutionary biologist and taxonomist for its distinctive characteristics in the Apiaceae family. Although some chloroplast genomes data have been now available, the changes in the structure of chloroplast genomes and selective pressure in the genus have not been fully understood. In addition, few of the species are endemic to Southwest China, a distribution and diversity center of Chinese *Bupleurum*. Endemic species are key components of biodiversity and ecosystems, and investigation of the chloroplast genomes features of endemic species in *Bupleurum* will be helpful to develop a better understanding of evolutionary process and phylogeny of the genus. In this study, we analyzed the sequences of whole chloroplast genomes of 4 Southwest China endemic *Bupleurum* species in comparison with the published data of 17 *Bupleurum* species to determine the evolutionary characteristics of the genus and the phylogenetic relationships of Asian *Bupleurum*.

**Results:**

The complete chloroplast genome sequences of the 4 endemic *Bupleurum* species are 155,025 bp to 155,323 bp in length including a SSC and a LSC region separated by a pair of IRs. Comparative analysis revealed an identical chloroplast gene content across the 21 *Bupleurum* species, including a total of 114 unique genes (30 tRNA genes, 4 rRNA genes and 80 protein-coding genes). Chloroplast genomes of the 21 *Bupleurum* species showed no rearrangements and a high sequence identity (96.4–99.2%). They also shared a similar tendency of SDRs and SSRs, but differed in number (59–83). In spite of their high conservation, they contained some mutational hotspots, which can be potentially exploited as high-resolution DNA barcodes for species discrimination. Selective pressure analysis showed that four genes were under positive selection. Phylogenetic analysis revealed that the 21 *Bupleurum* formed two major clades, which are likely to correspond to their geographical distribution.

**Conclusions:**

The chloroplast genome data of the four endemic *Bupleurum* species provide important insights into the characteristics and evolution of chloroplast genomes of this genu, and the phylogeny of *Bupleurum*.

**Supplementary Information:**

The online version contains supplementary material available at 10.1186/s12864-021-08008-z.

## Background

*Bupleurum* L., with more than 180 species, represents one of the largest genera of the family Apiaceae and is distributed in the north temperate zone (mainly in Eurasia, the Mediterranean, North Africa, Asia and North America) [1, 2]. Different from other genera of Apiaceae, life forms in *Bupleurum* vary greatly, ranging from herbs to shrubs [3]. The genus has frequently attracted the attention of evolutionary biologists and taxonomists for its distinctive characteristics in the Apiaceae family [1–6].

*Bupleurum* is easily distinguished from other genera of the family for its simple and entire leaves as well as conspicuous bracts and bracteoles, which are almost unique morphology characteristics in the family [1, 4]. Molecular phylogenetic studies in the recent two decades based on plastid and nuclear markers suggest that it should be considered as an identifiable tribe [7–12]. Interspecific phylogeny of *Bupleurum* presents a long standing problem in the systematics. The genus shows broad intra-specific morphological variations with poorly defined inter-specific boundaries, making the taxonomy based on traditional classification systems extremely difficult as increasing species being discovered [5, 6, 13]. In spite of the efforts at phylogenetic analysis in previous studies, some essential problems concerning the phylogenetic relationship of *Bupleurum* based on nrDNA (ITS) and various plastid sequences (e. g. *rps*16, *trn*H-*psb*A, and *mat*K) still remain to be solved [1, 4, 13, 14].

Compared with nuclear and mitochondrion genome, gene density of chloroplast genome is larger and the evolution rate is moderate, and the segments with different evolution rates may serve for different research purposes [15–19]. The evolution of chloroplast genomes has long fascinated and puzzled evolutionary biologists. The understanding of the evolutionary connection among the plant species, the features they shared, and their differences from other taxonomic groups [20] all benefit from comparative analysis of whole chloroplast genomes that provide insights into the phylogenetic relationships and species evolution in different taxa [21–28]. In general, chloroplast genome has long been considered to be conserved and affected little by adaptive evolution in many genera [18–20]. However, rearrangements, differences in structure, size, gene content and order, and positively selected genes have been documented in some genera, such as *Amphilophium* [29], *Amorphophallus* [30] and the apiaceous genus *Ligusticum* [31]. For such a diversified and wide-distributed genus like *Bupleurum*, we can not assert that there is absolutely no variation in chloroplast genome in a certain group without detailed study. It is important to investigate chloroplast genomes of the taxonomically significant genera, for understanding how infrageneric species are linked, what features are shared among them, and how they are different from other taxonomic groups [20, 29]. The advancement of the high-throughput sequencing technologies has drastically lowered the cost of analysis of the whole chloroplast genome sequences. Previous studies have reported the sequence data of chloroplast genomes for some species of *Bupleurum* [32–40], but unfortunately the changes in the structure of chloroplast genomes or the selective pressure in the genus were seldom addressed. As a result, the evolution of chloroplast of the genus is poorly understood. In the study by Li et al. [37], the chloroplast genomes of only five *Bupleurum* species were reported, but few of the species are endemic to Southwest China. Southwest China, which harbors an extremely high species diversity [41, 42] and is a distribution and diversity center of Chinese *Bupleurum* (ca. 21 species), including 12 species and 8 varieties endemic to China, 11 species and 5 varieties endemic to Southwest China. As key components of biodiversity and ecosystems, endemic species has long attracted the interest of ecologists and evolutionary biologists [43–47]. Investigation of the chloroplast genome features of endemic *Bupleurum* species may provide important insights into the evolution and phylogeny of the genus, especially the endemic *Bupleurum* species, thus helping to clarify the phylogenetic relationships and evolutionary aspects in the genus.

*Bupleurum shanianum*, *B. yunnanense*, B. *kweichowense* and *B. rockii* are endemic to Southwest China [2, 6]. *B*. *shanianum*, *B. yunnanense* and B. *kweichowense* are 3 perennial herbs with height ca. 6–35(− 58) cm, 12–35 cm, 20–40 cm, respectively, while *B. rockii* has a relatively higher height ca. 60–100 cm. *B*. *shanianum* and *B. yunnanense* grow among grassy places, bushes, or under forests at altitudes of 3200–4400 m and 2500–5000 m, respectively, distributed in the alpine area of eastern Himalayas (Southeast Tibet), Sichuan and Yunnan Provinces of China. *B. rockii* grows in open forests and grasses on mountain slopes at altitudes of 1900–4200 m, occurring in Sichuan and northwest Yunnan Provinces of China. The documentation of *B. kweichowense* is extremely poor and only a few collections from northeast Guizhou Province (Fanjing Shan) of China are available. It grows on gravelly slopes in sunny places at altitudes ca. 2100 m.

In this study, we used high-throughput sequencing technologies to sequence the chloroplast genomes of four *Bupleurum* species (*B. shanianum*, *B. yunnanense*, *B. kweichowense* and *B. rockii*) and assembled their whole chloroplast genomes. Previously published chloroplast genomes of 17 *Bupleurum* species [32–40], including herbs and one shrub (*B. dracaenoides*), were downloaded for comparative analysis, and the 21 species are distributed roughly in 3 regions (Southwest China; Northwest China; Northeast China, Korea and Japan; Additional file 1: Fig. S1 and Additional file 2: Table S1), which vary greatly in geomorphology and climate. Environmental heterogeneity plays an important role in evolutionary trajectories and ecological adaption of species. It has been reported that the positive selection on some plastid genes (e. g. *clp*P, *ndh*F and *mat*K) were observed on some plastid genes in some genera, which indicate that these genes might be subject to adaptive evolution in response to distinct ecological selective pressures [29–31]. However, to date, adaptive evolution of chloroplast genes in *Bupleurum* has not been fully understand. The sequence data of the chloroplast genomes provide a clue to the evolution and phylogeny of *Bupleurum*. Here, we attempted to answer the following questions: (1) Are there differences in the gene and structure of chloroplast genomes among *Bupleurum* species with different life forms and distributions? (2) Do the genes of *Bupleurum* species suffer positive selection under different habitats? We also constructed a phylogeny using 80 protein-coding genes (PCGs) of 21 *Bupleurum* species and 2 outgroups to explore the interspecific phylogenetic relationship of *Bupleurum* species in these regions.

## Results

### Chloroplast genome features of four *Bupleurum* species endemic to Southwest China

**Fig. 1:**
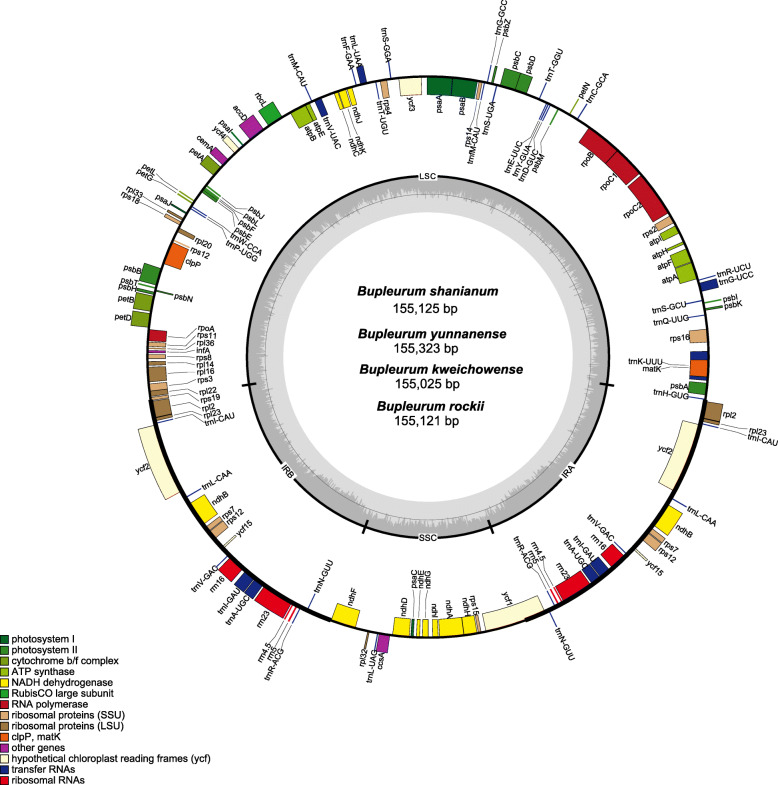
Chloroplast genome map of the four *Bupleurum* species. Genes shown outside of the larger circle are transcribed clockwise, while genes shown inside are transcribed counterclockwise. Thick lines of the smaller circle indicate IRs and the inner circle represents the GC variation across the genic regions

The sequences of the 4 *Bupleurum* chloroplast genomes ranged from 154,925 bp (*B*. *kweichowense*) to 155,323 bp (*B*. *yunnanense*), all having the typical quadripartite structure, comprising a SSC (17,478–17,575 bp) and a LSC (84,920–85,228 bp) region separated by a pair of IRs (52,572–52,649 bp) (Table 1, Fig. 1). The LSC regions exhibited the greatest standard deviation in sequence length (cv = 0.3%), followed by the IR regions (cv = 0.1%) and SSC regions (cv = 0.08%). The overall GC content was highly similar across the 4 chloroplast sequences (37.7–37.8%) (Table 1).

**Table 1:** Summary of chloroplast features in four *Bupleurum* species

	*B. shanianum*	*B. yunnanense*	*B. kweichowense*	*B. rockii*
Total length (bp)	154,925–155,258	155,211–155,323	155,025	155,120–15,121
Total GC content (%)	37.8	37.8	37.7	37.8
IRa length (bp)	26,261–26,271	26,212–26,279	26,299	26,322
IRb length (bp)	26,261–26,300	26,212–26,279	26,299	26,327
SSC length (bp)	17,478	17,487–17,530	17,575	17,515
LSC length (bp)	84,921–85,208	85,148–85,228	84,920	84,959–84,960
Number unique genes	114	114	114	114
Protein coding	80	80	80	80
tRNA genes	30	30	30	30
rRNA genes	4	4	4	4

The chloroplast gene contents of the 4 *Bupleurum* species were identical (Table 1). Each *Bupleurum* chloroplast genome encoded a total of 114 unique genes, consisting of 30 tRNA genes, 4 rRNA genes and 80 protein-coding genes (PCGs) with the same gene order (Table 1). The SSC region contained 11 PCGs (*ndh*F, *rpl*32, ccsA, *ndh*D, *psa*C, *ndh*E, *ndh*G, *ndh*I, *ndh*A, *ndh*H, and *rps*15) and 1 tRNA (*trn*L-UAG), while the LSC region contained 60 PCGs and 22 tRNAs (Table 2). A total of 20 genes were duplicated in the IR regions, including 8 tRNAs (*trn*A-UGC, *trn*G-UCC, *trn*I-GAU, *trn*I-CAU, *trn*L-CAA, *trn*N-GUU, *trn*R-ACG, and *trn*V-GAC), 8 PCGs (*rps*7, *rpl*2, *rpl*23, *ndh*B, *rps*7, *rps*12, *ycf*2 and *ycf*15), and 4 rRNAs (*rrn*4.5, *rrn*5, *rrn*16 and *rrn*23) (Table 2). Eighteen genes contain introns, 15 of which contain a single intron, whereas the other 3 (*clp*P, *ycf*3 and *rps*12) harbored 2 introns (Table 2). In this study, the incomplete copy of *ycf*1 and *rps*19 in the IR regions were regarded as pseudogenes.

**Table 2:** List of genes encoded in four *Bupleurum* plastomes

Gene Category	Genes	Number
Ribosomal RNAs	*rrn*4.5 ^(×2)^; *rrn*5 ^(×2)^; *rrn*16 ^(×2)^; *rrn*23 ^(× 2)^	4
Transfer RNAs	t*rn*A-UGC ^(×2)a^; *trn*C-GCA; *trn*D-GUC; *trn*E-UUC; *trn*F-GAA; *trn*fM-CAU; *trn*G-GCC; *trn*G-UCC ^a^; *trn*H-GUG; *trn*I-CAU ^(× 2)^; *trn*I-GAU ^(× 2) a^; *trn*K-UUU ^a^; *trn*L-CAA^(× 2)^; *trn*L-UAA ^a^; *trn*L-UAG; *trn*M-CAU; *trn*N-GUU ^(× 2)^; *trn*P-UGG; *trn*Q-UUG; *trn*R-ACG ^(× 2)^; *trn*R-UCU; *trn*S-GCU; *trn*S-GGA; *trn*S-UGA; *trn*T-GGU; *trn*T-UGU; *trn*V-GAC ^(× 2)^; *trn*V-UAC ^a^; *trn*W-CCA; *trn*Y-GUA	37
Subunits of photosystem I	*psa*A; *psa*B; *psa*C; *psa*I; *psa*J; *ycf*3^b^; *ycf*4	7
Subunits of photosystem II	*psb*A; *psb*B; *psb*C; *psb*D; *psb*E; *psb*F; *psb*H; *psb*I; *psb*J; *psb*K; *psb*L; *psb*M; *psb*N; *psb*T; *psb*Z;	15
ATP-dependent protease subunit P	*clp*P^b^	1
Large subunit of rubisco	*rbc*L	1
NADH dehydrogenase	*ndh*A; *ndh*B ^(×2) a^; *ndh*C; *ndh*D; *ndh*E; *ndh*F; *ndh*G; *ndh*H; *ndh*I; *ndh*J; *ndh*K	12
Ribosomal protein (large subunit)	*rpl*2 ^(×2) a^; *rpl*14; *rpl*16 ^a^; *rpl*20; *rpl*22; *rpl*23 ^(× 2)^; *rpl*33; *rpl*32; *rpl*36	11
Small subunit of ribosomal proteins	*rps*2; *rps*3; *rps*4; *rps*7 ^(×2)^; *rps*8; *rps*11; *rps*12 ^(× 2)b^; *rps*14; *rps*15; *rps*16 ^a^; *rps*18; *rps*19	14
DNA-dependent RNA polymerase	*rpo*A; *rpo*B; *rpo*C1; *rpo*C2	4
Subunits of ATP synthase	*atp*A; *atp*B; *atp*E; *atp*F ^a^; *atp*H; *atp*I	6
C-type cytochrome synthesis gene	*ccs*A	1
Subunits of cytochrome b/f complex	*pet*N; *pet*A; *pet*L; *pet*G; *pet*B ^a^; *pet*D ^a^	6
Envelop membrane protein	*cem*A	1
Maturase	*mat*K	1
Hypothetical chloroplast reading frames	*ycf*1; *ycf*2 ^(×2)^	5
Subunits of Acetyl-CoA-carboxylase	*acc*D	1
Pseudogenes	*inf*A; *rps*19^c^; *ycf*1^c^; *ycf*15 ^(×2)^	4
Total	114 single-copy genes, 132 in total.	

(×2): Two gene copies in the IRs; ^a^ Gene containing one intron; ^b^ Gene containing two introns; ^c^ means the incomplete copy located in the IR of the gene straddling the IR and LSC/SSC regions

### Comparative analysis of chloroplast genome structure of *Bupleurum*

**Fig. 2:**
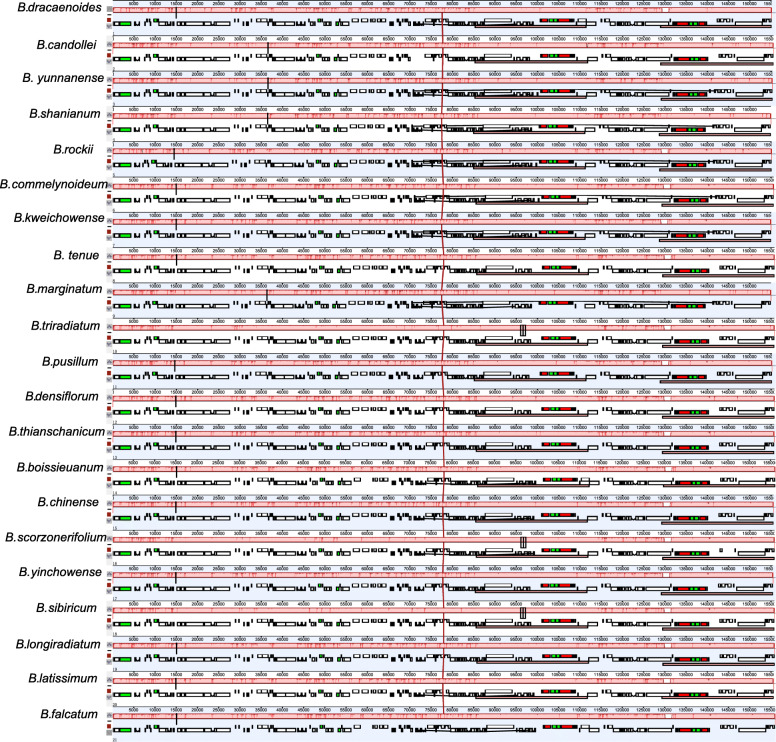
MAUVE alignment of 21 *Bupleurum* species chloroplast genomes using Geneious. Within each of the alignments, local collinear blocks are represented by blocks of the same color and linked

**Fig. 3:**
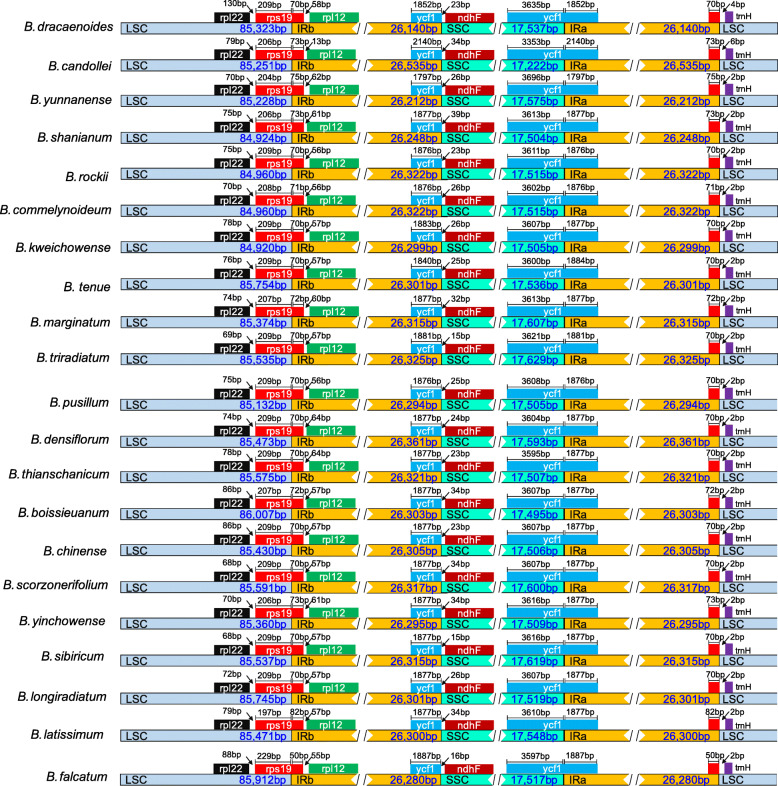
Comparison of the LSC, SSC and IR junction among the 21 *Bupleurum* cp genomes

The Mauve alignment analysis revealed that there was no rearrangement in coding and non-coding regions of the 21 *Bupleurum* chloroplast genomes (Fig. 2), indicating that the chloroplast genomes were conserved. Among the 21 *Bupleurum* species, the genes *rpl*22 and *rpl*2 flanked the LSC/IRb junction, and gene *rps*19 traversed the LSC and the IRb region (JLB line), with 50–82 bp occurring in the IR region (Fig. 3). On the other side of the IRb/SSC was the gene *ndh*F, which was 15–39 bp away from the IRb/SSC junction. The *ycf*1 gene traversed the SSC and IRa region, with 1797–2140 bp occurring in the IR region (Fig. 3).

Simple sequence repeats (SSRs) analysis showed that total number of SSR loci ranged from 59 (*B. dracaenoides*) to 83 (*B. thianschanicum*). The patterns of SSRs distribution were similar among the 21 *Bupleurum*, as shown in Additional file 1: Fig. S2. Mono-nucleotides were the most frequent in the SSRs (61.5–71.2%), followed by di-nucleotides (10.1–19.7%). Tri-nucleotides and tetra-nucleotides were more frequent than penta- and hexa-nucleotides (Additional file 1: Fig. S2). Short dispersed repeats (SDRs) analysis showed that total number of SDRs ranged from 34 (*B. candollei* and *B. shanianum*) to 49 (*B. longiradiatum*). The species of group I showed less SDRs than those of group II and III (Additional file 1: Fig. S3). The 21 *Bupleurum* species tended to generate more forward and palindromic repeats rather than reverse repeats, and lacked complement repeats (Additional file 1: Fig. S3).

### Genome divergent hotspot regions in *Bupleurum* species

Comparative sequence analysis of the 21 *Bupleurum* species using mVISTA revealed a high sequence identity across the 21 species (Additional file 1: Fig. S4), with identity ranged from 96.4 to 99.2%, suggesting that *Bupleurum* chloroplast genomes were fairly conserved. Overall, the coding regions (identity = 99.7 ± 0.8%) were less divergent than non-coding regions (identity: = 96.7 ± 4.1%), and the IR regions (identity = 99.5 ± 1.1%) were more conserved than LSC (identity = 97.1 ± 3.1%) and SSC (identity = 98.2 ± 2.4%) regions. Variations were observed in some intergenic spacers, including *trn*K-*rps*16, *rps*16-*psb*K, *trn*G-*trn*R, *atp*I-*rps*2, *trn*C-*trn*T, *pet*A-*psb*J, *psa*C-*ndh*G and *ycf*1-*trn*R. A few divergent regions were also observed in some coding regions including *psb*D, *atp*B, *ndh*D and *ycf*1.

The nucleotide diversity (Pi) of the chloroplast genomes in the 21 *Bupleurum* species was also calculated to assess the sequence divergence level of these species. In the LSC region, Pi values averaged 0.01087 (range 0.00063–0.03092), and in the SSC region, the average value was 0.01527 (range 0.00394–0.03107); the IR region had the lowest average value of 0.00215 (range 0–0.00963) (Additional file 1: Fig. S5). Most of the sequences with high Pi values were spacer regions between genes. Among these spacer regions, *trn*K-*rps*16, *rps*16-*psb*K, *trn*G-*trn*R, *atp*I-*rps*2, *trn*C-*trn*T, *pet*A-*psb*J, *psa*C-*ndh*G and *trn*R-*ycf*1 were the 8 regions having Pi values > 0.02000. Only 4 coding regions (*psb*D, *atp*D, *ndh*D and *ycf*1) had high Pi values over 0.02000.

### Selective pressure analysis

**Fig. 4:**
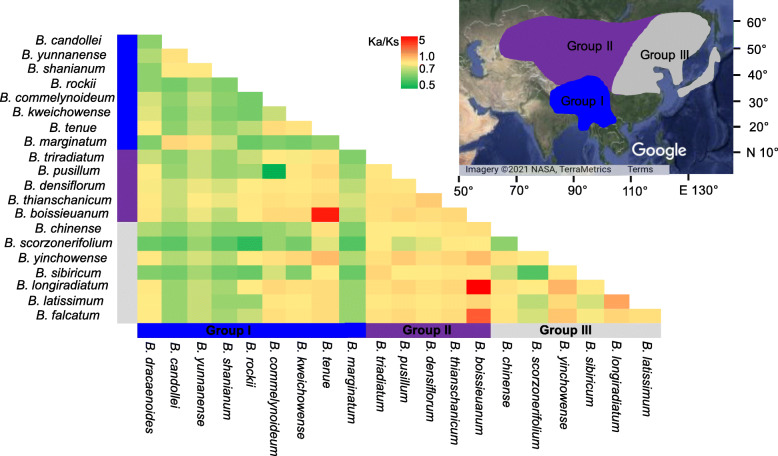
Pairwise Ka/Ks ratios in *Bupleurum*. This heatmap shows pairwise Ka/Ks ratios between every sequence in the multigene nucleotide alignment. The original satellite imagery was obtained from Google Maps (Map data: Google, TerraMetrics; https://maps.google.com/), and modified with Adobe Illustrator CS6 (Adobe Systems Incorporated, San Jose, CA, USA)

The rate of synonymous substitutions and non-synonymous substitutions (Ka/Ks) of 80 protein-coding genes were calculated to assess the selection pressure between *Bupleurum* species. At the species level, by concatenating all of the 80 genes into a super-matrix, the Ka/Ks ratios ranged from 0.50 (*B. commelynoideum* vs *B. pusillum*) to 5.0 (*B. longiradiatum* vs *B. boissieuanum*), with an average ratio of 0.92 (Fig. 4). The Ka/Ks ratios between group II and group III were the highest (averaging 1.10) (Additional file 2: Table S2). The Ka/Ks ratios within group II (averaging 0.98) and group III (averaging 0.92) were higher than those between group I and group II, (averaging 0.83), between group I and group III (averaging 0.71), and within group I (averaging 0.69) (Additional file 2: Table S2).

The Ka/Ks ratios were also calculated for all the 80 protein-coding genes in the chloroplast genomes of the 21 *Bupleurum* species separately (Additional file 2: Fig. S6 and Fig. S7). Among the genes, *mat*K had highest Ka/Ks ratios (around 1.0, especially in group II), following by *ycf*2, *acc*D, and *clp*P. The remainder had Ka/Ks ratios ranged from 0 to 0.6. The mean Ka/Ks ratios of protein-coding genes in LSC (0.10) and SSC regions (0.15) were lower than those in the IR regions (0.19). After sorting the genes into functional categories and groups, Ka/Ks of the photosynthetic genes (0.0412 ± 0.0683) were lower than those of genes related to self-replication (0.2507 ± 0.2197) as well as other genes (0.2065 ± 0.1812).

### Phylogenetic analysis

**Fig. 5:**
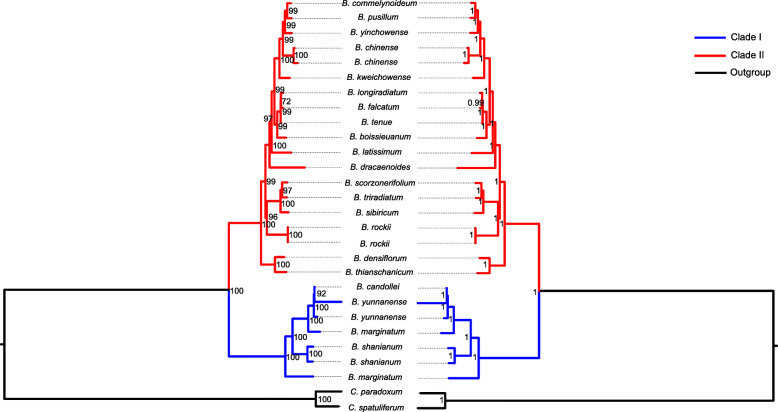
Molecular phylogenetic trees of 21 *Bupleurum* and 2 *Chamaesium* constructed by Maximum likelihood (left) and Bayesian inference (right) analysis based on 80 coding genes. Bootstrap support values (> 50%) based on maximum-likelihood (ML) and posterior probabilities analysis are labelled at each node

Results of Bayesian and ML analyses of the 21 *Burpleurum* and 2 outgroup chloroplast genomes are presented in Fig. 5. The phylogenic trees estimated from the Bayesian and ML analyses showed congruence in their topologies, high bootstrap support values (BS > 90%) and strong posterior probabilities (PP = 1) for most of the nodes. The two phylogenetic trees highlighted two clades (clade I and II), containing 4 (PP = 100, BS = 100%) and 17 species (PP = 100, BS = 100%), respectively.

## Discussion

### Differences in gene and structure of chloroplast genomes among *Bupleurum* species

We for the first time described the chloroplast genomes of the 4 *Bupleurum* species endemic to Southwest China, which provide important insights into the characteristics of plastid genomes of the members of this genus. Previous studies have suggested that the *Bupleurum* is a monophyletic group based on morphology and molecular (nrDNA and chloroplast fragments) evident. In present study, we provide new insight into the evolution of *Bupleurum* in term of chloroplast genomes [1, 4, 7–14]. Our results showed that the chloroplast genomes of *Bupleurum* species are extremely similar, indicating their high level of conservation especially in terms of chloroplast genome organization, tendency of SDRs and SSRs, gene content. The results support that *Bupleurum* is a monophyletic group in the aspect of chloroplast genomes as they are remarkably conserved.

The chloroplast genomes of the 21 *Bupleurum* species displayed the typical quadripartite structure, comprising a pair of IR regions which were separated by a LSC and a SSC region. The chloroplast genomes size of *Bupleurum*, ranging from 154,925 to 156,108 bp, is larger than those of the other genera in the Apiaceae family (e. g. *Angelica*, *Arracacia*, *Coriandrum*, *Glehnia*, *Heracleum*, *Ligusticum*, *Ostericum*, *Pastinaca*, *Pimpinella*, *Saposhnikovia*, *Semenovia*, *Seseli*, *Tetrataenium* [45–48]). The chloroplast genomes of the *Bupleurum* species showed only minor differences (~ 1 kb) in sizes. Previous studies suggested that the size variations of the chloroplast genomes resulted from expansion and contraction of the IR regions [20, 24, 48–50]. The IR boundary comparative analysis revealed that gene distributions at SC/IR junctions of *Bupleurum* chloroplast genomes were almost identical, and only minor differences were found in length of these genes (*rps*19 and *ycf*1) and SC/IR borders. However, the LSC/IR borders showed differences among *Bupleurum* species and some Apiaceae taxa. For instance, while the LSC/IRb junction was located within the *rps*19 gene in *Bupleurum* species as well as some taxa in Apiaceae (*Anthriscus*, *Daucus*, *Tiedemannia*, [48]), it resided within the *rps*12 gene in *Anethum* [48], *Foeniculum* [48], *Prangos* [51, 52], *Petroselinum* [48, 52]. Our repetitive sequences analysis in addition to previous studies [32–40] showed that the tendency of SDRs and SSRs were similar among *Bupleurum* species, while the certain diversity in numbers varied.

The gene/intron content and relative gene positions were highly conserved in *Bupleurum* species and almost identical to those in other members of the Apiaceae family [48, 51–56]. Two genes were found to be pseudogenes in *Bupleurum* species. The genes *ycf*1 and *rps*19, located in the IRa/SSC and LSC/IRb boundaries, respectively, were identified as pseudogenes on account of an incomplete duplication of the normal functional copy.

### High variable regions for potential molecular markers

Most *Bupleurum* species have important pharmaceutical values and their accurate identification is crucial to their utilization. However, the morphological similarities of the *Bupleurum* species make their authentication extremely difficult. With the development of the molecular technology, many of the chloroplast genome regions, especially the mutational hotspots (e.g. *ndh*F, *mat*K, *trn*S-*trn*G), have been widely implemented [21, 22, 57], while none of the commonly used region in chloroplast genome were effective for identification for different plant taxa.

In a comparative analysis of the complete plastid genome of 5 *Bupleurum* species, only 8 highly variable regions (Pi > 0.01) (e. g. *pet*N-*psb*M, *rbc*L-*acc*D and *ccs*A-*ndh*D) were identified, and all of them were spacer regions between genes [37]. In the present study, we obtained a different result with the supplement of another 17 *Bupleurum* species. The results of mVISTA and slide window analysis showed that the IR regions were more conservative and less variable than the SC regions, possibly as a result of copy number differences in the inverted repeats sequences caused by gene conversion [58]. Different from the findings by Li et al. [37], we identified 4 coding regions (*psb*D, *atp*D, *ndh*D and *ycf*1) with high Pi values (> 0.02) and the 17 regions reported by Li et al. [37] were not found to be the most variable regions in this study. Instead, another 8 intergenic regions (*trn*K-*rps*16, *rps*16-*psb*K, *trn*G-*trn*R, *atp*I-*rps*2, *trn*C-*trn*T, *pet*A-*psb*J, *psa*C-*ndh*G and *trn*R-*ycf*1) were identified, which contained frequent interspecies mutation (Pi > 0.02). As more species were included in our analysis, we propose that the 4 genes and 8 intergenic regions we identified can have much potentially to serve as high-resolution DNA barcodes in species authentication of *Bupleurum*.

### Do the genes of *Bupleurum* suffer positive selection under different habitats?

Plants are subjected to different selection pressures due to different types of stresses in varied habitats, and genes related to a specific environment are usually assumed to be under positive selection [59]. The *Bupleurum* species growing at different latitudes and different altitudes are exposed to different light intensities and temperature, and positive selection is likely to occur among these species in different regions.

Our results indicated that *Bupleurum* species under different habitats suffer positive selection, especially in Northeast China and Northwest China. We found that some genes, including *mat*K, *ycf*2, *acc*D, and *clp*P, were subjected to positive selection, suggesting that adaptive changes may have occurred more frequently in response to the highly selective conditions in different habitats. These genes have also previously been found under positive selection [60–62]. The *clp*P gene is essential for plant cells and encodes *clp*P proteases that degradants polypeptides [63]. The *mat*K gene is one of the fastest evolutionary genes, which functions in light-regulated activities and plant development [64, 65]. The *acc*D gene encodes the β-carboxyl transferase subunit of Acetyl-CoA carboxylase that is essential for the synthesis of products required for the extraplastidic processes needed for leaf development [66]. Gene *ycf*2 encodes products that are essential for cell survival [67] and chloroplast protein import [68]. These positively selected genes may contribute to the adaptation of species in *Bupleurum* to various environments. However, our analyses also showed strong purifying selection on most of the genes. Previous studies suggest that purifying selection acting on the genes generally leads to low synonymous and non-synonymous DNA substitution rates in chloroplast genomes of angiosperms, such as Araceae [69, 70] and Liliaceae [71, 72]. Purifying selection, one of the most prevalent mechanisms in natural selection, constantly eliminates deleterious mutations [73]. Most of the genes in these chloroplast genomes were thus subjected to extensive purifying selection to retain conserved functions in *Bupleurum*. The distribution of Ka/Ks indicated that most of the genes in the SSC region have experienced higher selection pressures than those in other chloroplast genome regions, whereas the IR region is more conserved. In addition, the genes involved in photosynthesis tend to have lower rates of evolution than genes related to self-replication and other functions. These differences are likely the results of variations in generation time, gene expression level, gene function, lengths of the encode protein products, and relaxed selection [74–77].

### Phylogenetic inference from plastid genomes of the genus *Bupleurum* in East Asia

Currently there is no widely acceptable infrageneric classification system of *Bupleurum* [78–80]. East Asian, especially China, is one of the diversity center for the genus *Bupleurum*. *Bupleurum* species in these regions are diverse, exhibiting various life forms (including herbs and shrubs) and pollen types (e. g. subrhomboid, subspheroid and subellipsoid) [40, 81]. However, there are gaps in phylogenetic relationships of East Asian *Bupleurum*, only a few efforts have been made based on morphology [81] and several DNA fragments [4, 13, 14]. Shu et al. proposed to divide Chinese *Bupleurum* into monotypic subgenus *Longifolia* (Wollf) Yuan and subgenus *Eubupleura* (including section *Falcata* and section *Ranunculoidea*) [81], while Wang et al. [4] did not support this treatment and proposed to divide the genus *Bupleurum* into two clades corresponding to the two subgenera (subg. *Penninervia*, subg. *Bupleurum*) of Neves and Watson [1]. The 21 *Bupleurum* species here have various life forms and widely distribute throughout East Asia (Southwest China; Northwest China; Northeast China, Korea and Japan), providing an opportunity to study the phylogenomic relationship in East Asian *Bupleurum* species. Based on the results of phylogenetic analysis, we propose to divide the 21 *Bupleurum* species, which were all from Asia, into two clades (clade I and clade II). The phylogenetic relationships within *Bupleurum* based on chloroplast genomes we presented herein are largely congruent to that of Wang et al. [4, 13] with only a few differences. *B. rockii* occurred in clade I in our study, but in another clade by Wang et al. [3, 13]. The two clades are likely to correspond to their geographical distribution, not the characteristics of bracteoles. The clade I consists of 4 species from Southwest China, with two endemic species to Southwest China (*B*. *shanianum* and *B*. *yunnanense*) and two species (*B. marginatum* and *B. candollei*) with extended distribution in such Himalayan countries as Bhutan, India, Kashmir, Myanmar, Nepal, Pakistan and Sikkim. The species in the clade II, except for *B*. *dracaenoides*, *B. rockii* and *B. kweichowense*, occur at higher latitudes of China such as Northwest and North China, Japan and Korea. Our results do not fully support Shu et al.’s treatment on *Bupleurum* [81], which divided the subg. *Eubupleura* into sect. Ranunculoidea and sect. Falcata based on the difference of bracteoles characteristic. However, the subgenus subdivision of the genus *Bupleurum* remains unresolved in this present study because of the lack of chloroplast genome data from subg. *Penninervia*, most species of which are distributed in the Mediterranean region. To access a complete reassessment of the interspecific relationship of *Bupleurum*, more complete plastid genomes information is required, especially the data of subg. *Penninervia*.

## Conclusion

In this study, we for the first time described 7 full chloroplast genomes of 4 *Bupleurum* species endemic to Southwestern China. Comparative analysis of chloroplast genomes of the 4 species against the published data of 17 *Bupleurum* species revealed that the chloroplast genomes of *Bupleurum* species were extremely conserved with similarities in terms of chloroplast genome organization, tendency of SDRs and SSRs, and gene content. In spite of the highly conservation of the chloroplast genomes of the *Bupleurum* species, some mutational hotspots have been detected, which can be potentially used as high-resolution DNA barcodes in discrimination of *Bupleurum* taxa. *Bupleurum* species under different habitats suffer positive selection, and some genes (*mat*K, *ycf*2, *acc*D, and *clp*P) are also subjected to positive selection. Phylogenetic analysis revealed that the 21 *Bupleurum* formed two clades, which are likely to correspond to their geographical distribution. The chloroplast genomes information reported herein and the comparative analysis of *Bupleurum* chloroplast genomes provide important insights into the evolution of the chloroplast genomes and phylogeny of *Bupleurum*.

## Methods

### Sample collection

Leaf from a total of 7 individuals of the 4 *Bupleurum* species (two individuals for each of *B. shanianum*, *B. yunnanense*, *B. rockii*, while one for *B. kweichowense*) were collected from the wild and were dried with silica gel, and stored at − 80 °C until required for DNA extraction. Voucher specimens were collected for each samples and deposited at the herbarium of Southern Medical University. All species were authenticated by prof. Zhi Chao (Southern Medical University). Details information of the samples and voucher numbers of the specimens were shown in Additional file 2: Table S3.

### DNA extraction, library construction and sequencing

Total DNA was extracted from silica-dried leaf material using a modified extraction method described by Yang et al. [82]. The quality and concentration of the extracted DNA were detected by 1.0% agarose gel electrophoresis and by a NanoDrop 2000C spectrophotometry (Thermo Fisher, US). The extraction genomic DNA (approximately 1 μg) was subjected to random degradation by Covaris (E210), and then fragments with a size of 200–400 bp were selected by using Agencourt AMPure XP-Medium kit. The selected fragments were amplified after suffering from end repair, 3′-adenylation and adaptor ligation. Then, they were heat denatured to single strand after purification. The single strands were circularized, and single strand circle DNA was obtained as the final library. The final library was sequenced by BGISEQ-500 (BGI, Shenzhen, China) to generate raw reads. The details of the quantity and quality of raw reads, and coverage depth of the assembled genomes were provided in Additional file 2: Table S4.

### Genome assembly and annotation

The generated raw sequencing data was filtered using program SOAPnuke [83] with default parameters to remove adapters, low-quality reads with quality value ≤10, to final obtain high-quality reads. Then, the high-quality reads were aligned to the published *B. commelynoideum* chloroplast genome (NCBI accession MT162552) using Geneious v 10.2.2 [84] with default settings. Subsequently, the matched reads were selected for de novo assembled with SPAdes v3.11.1 [85]. The accuracy of assembly was evaluated by detecting the sequence coverage and the reading segment coverage at the contig connection.

The assembled chloroplast genome annotations were annotated using GeSeq [86] with the reference chloroplast genome of *B*. *commelynoideum* (NCBI accession MT162552). All the tRNAs were scanned with tRNAscan-SE [87] and ARAGORN [88]. The visual presentations of the physical circular maps of the genomes were generated using OGDRAW [89]. Finally, the annotated chloroplast genomes of the 4 *Bupleurum* species were submitted to the National Center for Biotechnology Information database (NCBI) under the accession numbers MW135450-MW135456, which were listed in Additional file 2: Table S3.

### Genome structure and comparative analysis

First, the chloroplast genome characteristics of the 4 *Bupleurum* species were described. Therein, Geneious R8.1 [90] was employed to conduct the GC content. MISA-web (https://webblast.ipk-gatersleben.de/misa/) was implemented to search simple sequence repeats (SSRs) with minimum numbers of 10, 5, 4, 3, 3, 3 repeat units for mono-, di-, tri- tetra-, penta-, and hexa-nucleotide SSRs, respectively. Short dispersed repeats (SDRs) analysis was implemented in REPuter [91] with the following parameters: a minimal repeat size of 30 bp, and sequence identity≥90% (hamming distance of 3 kb). To determine the IR expansion/contraction, genes distributed in and beside the borders of LSC, SSC and IR regions were compared.

In order to examine the divergence hotspots among the *Bupleurum* species, the whole chloroplast sequences of the 4 *Bupleurum* species, together with 17 published chloroplast genomes of *Bupleurum* species downloaded from NCBI database (Additional file 2: Table S5), were aligned using Geneious software. Subsequently, they were compared and visualized using mVISTA (http://genome.lbl.gov/vista/mvista/submit.shtml) with the reference chloroplast genome sequence of *B*. *commelynoideum* (NCBI accession MT162552). DnaSP v. 6.0 was used for sliding window analysis for computing nucleotide diversity (pi) among the chloroplast genome sequences [92], with 600 bp windows size and 200 bp step size.

Selective pressure estimation for the 21 *Bupleurum* species was carried out by calculating the ratio of the non-synonymous substitution (Ka) to the synonymous substitution rate (Ks) for all protein-coding genes sequences in DnaSP v6.

### Phylogenetic analysis

In order to gain insight into the phylogeny of East Asian *Bupleurum*, a total of 19 available chloroplast complete genomes of 17 *Bupleurum* species were downloaded from the NCBI database (Additional file 2: Table S4). The chloroplast genomes data of two *Chamaesium* species (*C. paradoxum*, NCBI accession MK780227; *C. spatuliferum*, NCBI accession MN119371), belonging to subfamily Apioideae, were also downloaded and set as outgroups (Additional file 2: Table S4). Only a dataset of 80 protein-coding genes (PCGs) was used for the phylogenetic analyses. A 23-taxon sequence matrix including two outgroups were aligned using the Geneious software.

Phylogeny was conducted through two approaches, namely the Maximum likelihood (ML) analyses and a Bayesian inference (BI) analyses. ML phylogenetic analysis was performed in RAxML v8.2.4 [93]. First, the best likelihood tree was obtained from 100 starting trees using rapid bootstrap analyses with 1000 replicates. Multiparametric bootstrapping analyses with 1000 replicates was conduct to obtained the bootstrap for each node. Substitution model for the two analyses were GTRGAMMA model. Bayesian inference (BI) was conducted in MrBayes v3.2.6 [94, 95]. The best-fit nucleotide substitution model (GTR + I + GAMMA) for Bayesian analysis was inferred from jModelTest v. 2.1.10 under the Akaike information criteria (AIC) [96]. Markov chain Monte Carlo (MCMC) analysis was performed with 50 million generations and sampling trees every 5000 generations. The first 10% of trees were discarded as burn fraction, and the remaining trees were combined to synthesized the consistent tree and estimate posterior probabilities. The resulting trees were rooted with *C. paradoxum* and *C. spatuliferum* and visualized with FigTree v 1.4 (http://tree.bio.ed.ac.uk/software/fgtree/).

## Supplementary Information

**Additional file 1.**

**Additional file 2.**

## Data Availability

Sequence information of the 7 chloroplast genomes of the four *Bupleurum* species (*B. shanianum*, *B. yunnanense*, B. *kweichowense* and *B. rockii*) is available in the National Center for Biotechnology Information database (NCBI) under the accession number MW135450-MW135456 (Additional file 2: Table S3). The accession numbers corresponding to the additional datasets used and analysed in this study can be found in Additional file 2: Table S5. These additional data were retrieved from the National Center for Biotechnology Information database (https://www.ncbi.nlm.nih.gov/).

## Abbreviations

ITS: Internal transcribed spacer region
nrDNA: Nuclear ribosome DNA
NGS: Next-generation sequencing
Cp: Chloroplast
LSC: Large single copy
SSC: Small single copy
IR: Inverted repeat
tRNA: Transfer RNA
rRNA: Ribosomal RNA
SSR: Simple sequence repeat
SDR: Short dispersed repeat
Met: Methionine
Trp: Tryptophan
Arg: Arginine
Leu: Leucine
Ser: Serine
RSCU: Synonymous codon usage
GC3: GC content on the third synonymous codon position
Ks: Rate of synonymous substitutions
Ka: Rate of non-synonymous substitutions
Pi: Polymorphic information
ML: Maximum likelihood
BS: Bootstrap support value
PP: Posterior probability
BI: Bayesian inference
AIC: Akaike information criteria
MCMC: Markov chain Monte Carlo.
