# Supplementary material for: The chloroplast genomes of four Bupleurum (Apiaceae) species endemic to Southwestern China, a diversity center of the genus, as well as their evolutionary implications and phylogenetic inferences
Source: BMC Genomics. 2021 Oct 2;22:714. doi: 10.1186/s12864-021-08008-z (PMC8487540; doi:10.1186/s12864-021-08008-z)
Supplement: Supplementary file 1 — Additional file 1. [file 12864_2021_8008_MOESM1_ESM.docx]

**
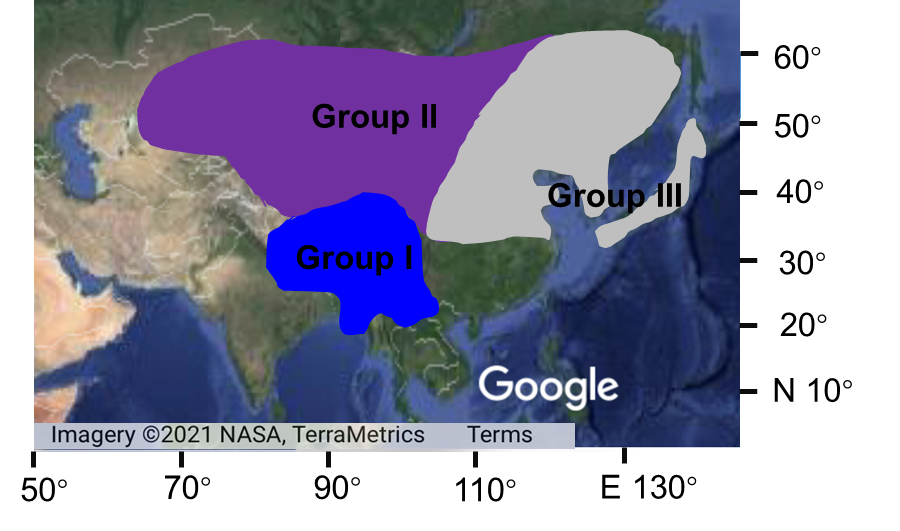
**

**Fig. S1** Distribution of three groups of *Bupleurum*. Group I include *B. dracaenoides*, *B. candollei*, *B. yunnanense*; *B. shanianum*, *B. rockii*, *B. commelynoideum*, *B. kweichowense*, *B. tenue*, *B. marginatum*; Group II include *B. triradiatum*, *B. pusillum*, *B. densiflorum*, *B. thianschanicum*, *B. boissieuanum*; Group III include *B. chinense*, *B. scorzonerifolium*, *B. yinchowense*, *B. sibiricum*, *B. longiradiatum*, *B. latissimum*, *B. falcatum*. The original satellite imagery was obtained from Google Maps (Map data: Google, TerraMetrics; https://maps.google.com/), and modified with Adobe Illustrator CS6 (Adobe Systems Incorporated, San Jose, CA, USA).

Fig. S2
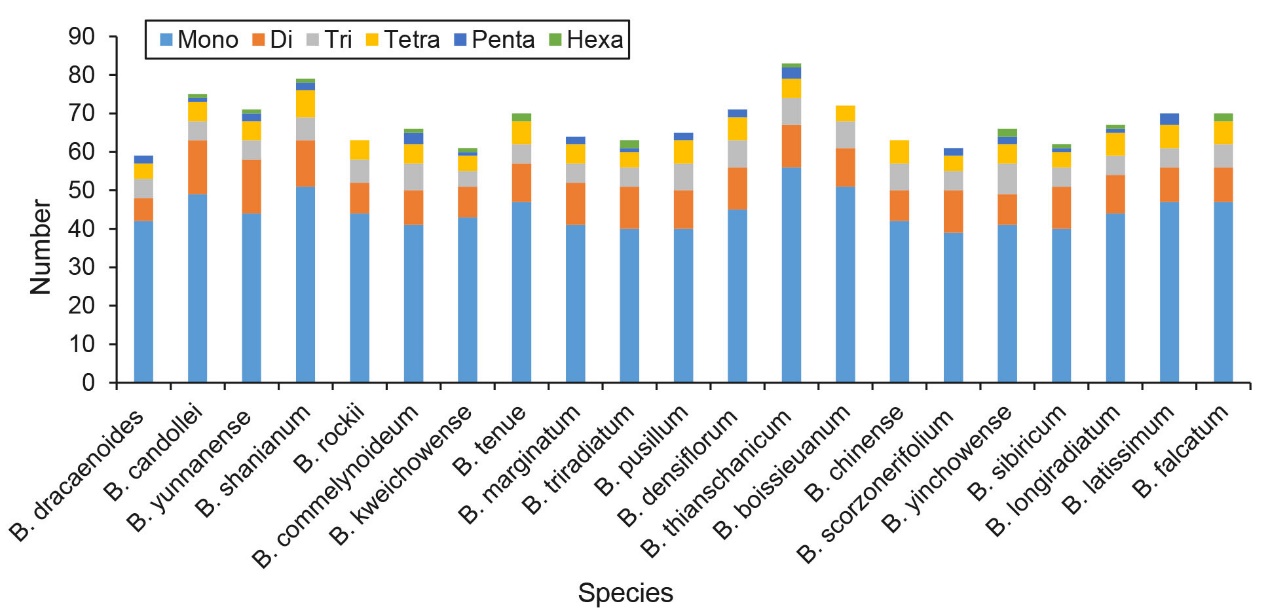
Simple sequence repeats (SSRs) in the 21 *Bupleurum* cp genomes.


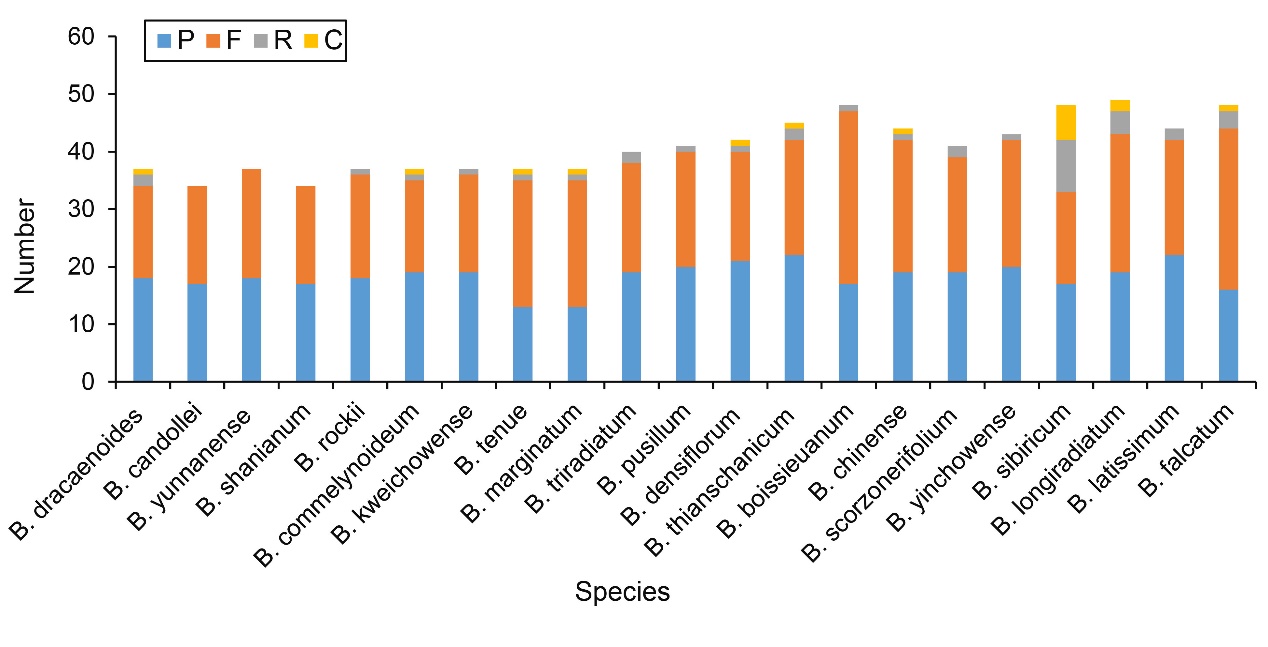
Fig. S3 Numbers of four types short dispersed repeats (SDRs) in the 21 Bupleurum species.


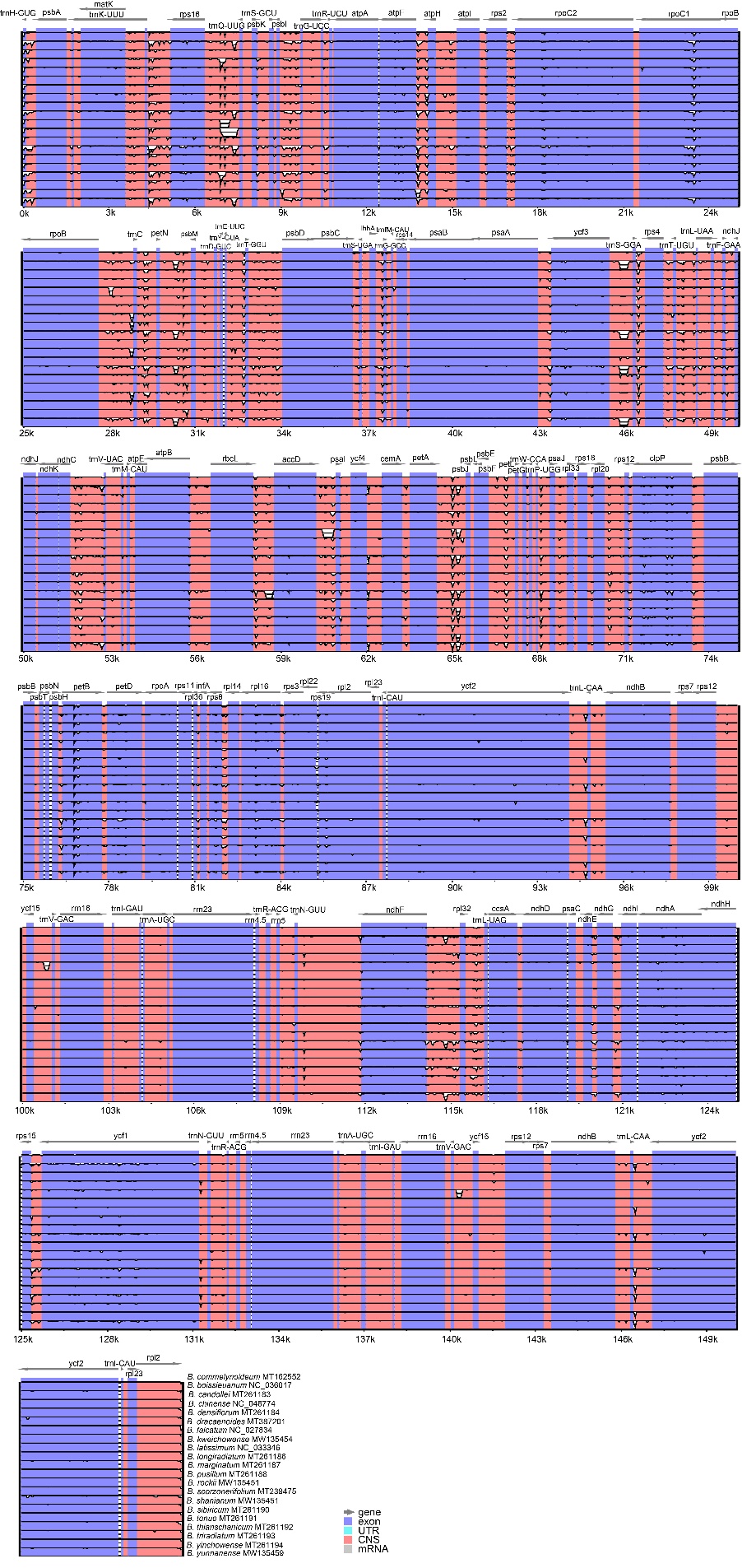
Fig. S4 Plots of percent sequence indentity of the chloroplast genomes of 21 *Bupleurum* species with *B*. *commelynoideum* (NCBI accession number: MT162552) as a reference. The percentage of sequence identities were estimated and plots were visualized in mVISTA.


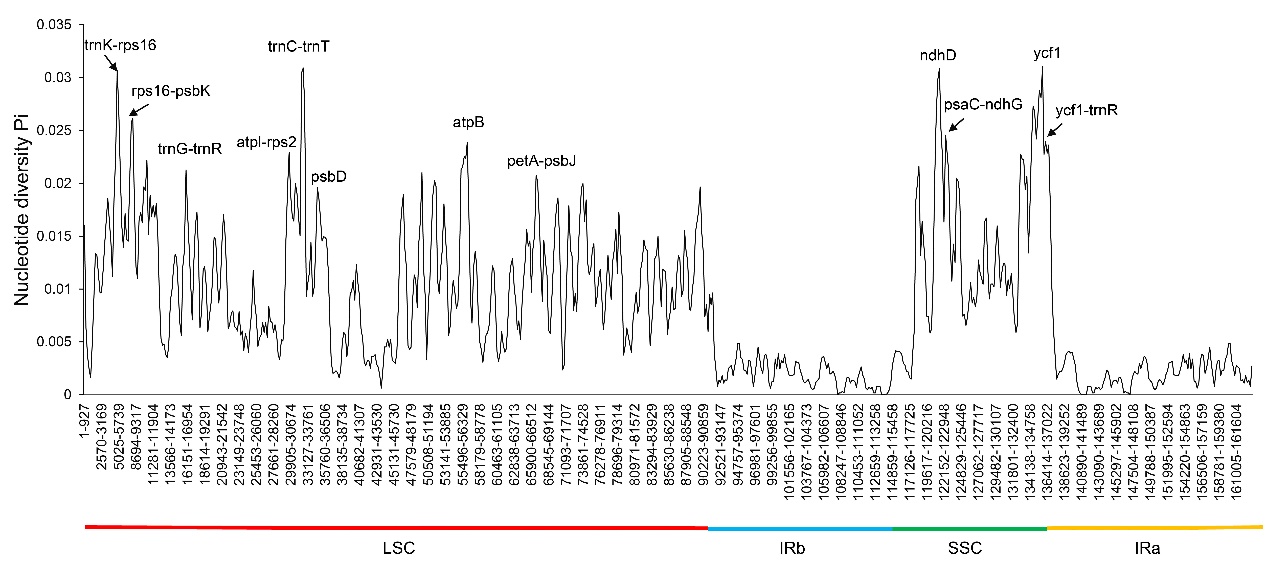
Fig. S5 The nucleotide diversity of the chloroplast genomes of the 21 *Bupleurum* species. Twelve regions with the highest Pi values were marked out. LSC: large single-copy region; IR: inverted repeats region; SSC: small single-copy region.


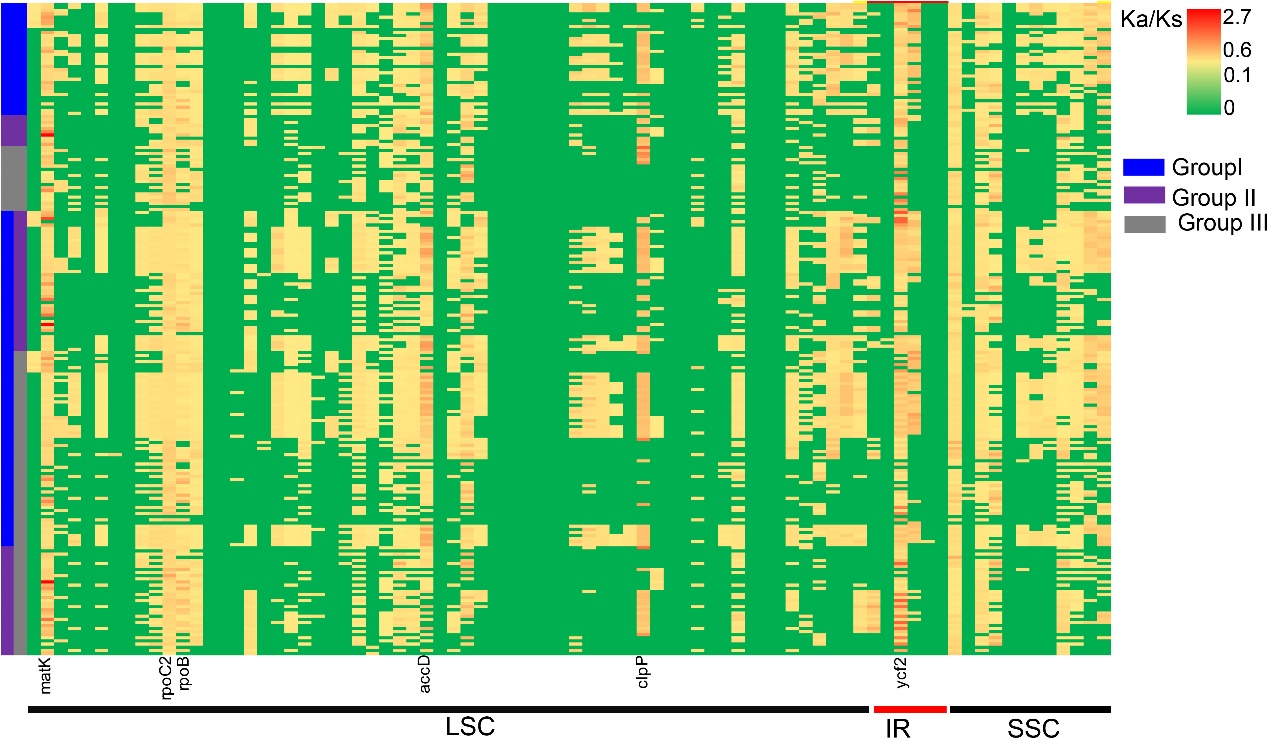
Fig. S6 Pairwise Ka/Ks ratios in *Bupleurum* in different genes. This heatmap shows pairwise Ka/Ks ratios among each individual gene in the *Bupleurum* species.


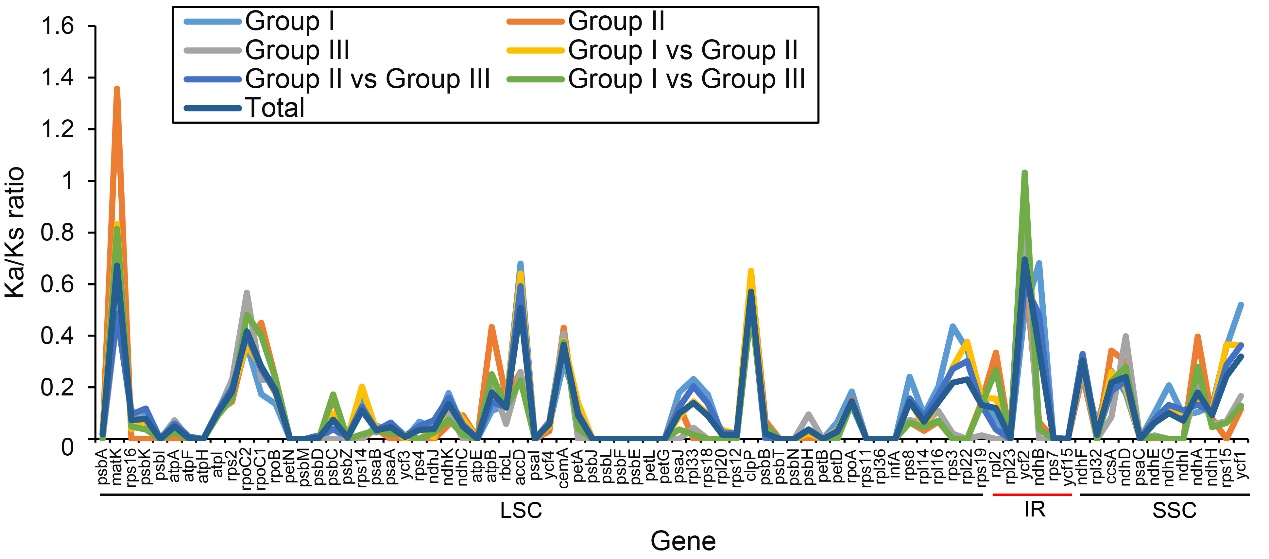


Fig. S7 Selective pressure of 80 protein-coding genes in *Bupleurum* species.
