# Supplementary material for: The chloroplast genomes of four Bupleurum (Apiaceae) species endemic to Southwestern China, a diversity center of the genus, as well as their evolutionary implications and phylogenetic inferences
Source: BMC Genomics. 2021 Oct 2;22:714. doi: 10.1186/s12864-021-08008-z (PMC8487540; doi:10.1186/s12864-021-08008-z)
Supplement: Supplementary file 2 — Additional file 2. [file 12864_2021_8008_MOESM2_ESM.docx]

**Table S1** Summary of distribution of 21 *Bupleurum* species included in present study.

| Group set | Species | Distribution |
| --- | --- | --- |
| Group I  (Mainly distributed in southwest China) | *B. dracaenoides* | Northern Yunnan and western Sichuan of China. |
|  | *B. candollei* | Yunnan, west Sichuan and south Xizang of China, Bhutan, North India, Kashmir, North Myanmar, Nepal, Pakistan, Sikkim. |
|  | *B. yunnanense* | Yunnan, Sichuan and Southeast Xizang of China. |
|  | *B. shanianum* | Eastern Himalayas, western Sichuan and northwestern Yunnan (Baima mountains) of China. |
|  | *B. rockii* | Northwest Yunnan and Sichuan of China. |
|  | *B. commelynoideum* | Northwest Yunnan, west Sichuan, Xizang South Gansu and Southeast Qinghai of China. |
|  | *B. kweichowense* | Northeast Guizhou of China. |
|  | *B. tenue* | Northwest Yunnan, Sichuan, Guizhou, Soth Xizang, Hubei and Guangxi of China, Bhutan, North India, Kashmir, Malaysia, Myanmar, Nepal, Pakistan, Sikkim, Thailand, Vietnam |
|  | *B. marginatum* | Yunnan, Sichuan, Guizhou, Xizang,Gansu, Hubei, Qinghai of China, Bhutan, Northeast India, Kashmir, Myanmar, Nepal, Pakistan, Sikkim. |
|  |  |  |
| Group II  (Mainly distributed in northwest China) | *B. triradiatum* | Norhwest Yunnan, west Sichuan, Xizang, Xinjiang and Qinghai of China |
|  | *B. pusillum* | Nei Mongol, Ningxia, Qinghai and Xinjiang of China, Mongolia, Russia. |
|  | *B. densiflorum* | Qinghai and Xinjiang of China, Kazakhstan, Kyrgyzstan, Tajikistan |
|  | *B. thianschanicum* | West Xinjiang of China, Kazakhstan, Kyrgyzstan. |
|  | *B. boissieuanum* | Sichuan, Gansu, Henan, Hubei and Shaanxi of China. |
|  |  |  |
| Group III  (Mainly distributed in north and northeast China, Japan and Korea) | *B.* *chinense* | Anhui, Gansu, Hebei, Heilongjiang, Henan, Hubei, Hunan, Jiangsu, Jiangxi, Jilin, Liaoning, Nei Mongol, Shaanxi, Shandong, Shanxi and Zhejiang of China |
|  | *B. scorzonerifolium* | Anhui, Gansu, Guangxi, Hebei, Heilongjiang, Jiangsu, Jilin, Liaoning, Nei Mongol, Shaanxi, Shandong and Shanxi of China, Japan, Korea, Mongolia, Russia. |
|  | *B. yinchowense* | Gansu, Nei Mongol, Ningxia and Shaanxi of China |
|  | *B. sibiricum* | Hebei, Heilongjiang, Liaoning and Nei Mongol of China, Mongolia, Southeast Russia. |
|  | *B. longiradiatum* | Gansu, Heilongjiang, Jilin, Liaoning and Nei Mongol of China, Japan, Korea, Southeast Russia. |
|  | *B. latissimum* | Korea |
|  | *B. falcatum* | Japan |

**Table S2** Minimum values, maximum values and mean values of pairwise Ka/Ks ratios within and between groups in Bupleurum.

|  | Min | Max | Mean |
| --- | --- | --- | --- |
| Group I | 0.59 | 1.10 | 0.69 |
| Group II | 0.82 | 1.30 | 0.98 |
| Group III | 0.57 | 2.00 | 0.92 |
| Group I vs Group II | 0.50 | 4.50 | 0.83 |
| Group I vs Group III | 0.54 | 1.60 | 0.71 |
| Group II vs Group III | 0.67 | 5.00 | 1.10 |

**Table S3** Details of samples of the four *Bupleurum* species.

| **Species** | **Location** | **Latitude (°N)** | **Longitude (°E)** | **Altitude (m)** | **Vouchers** | **GenBank accession** |
| --- | --- | --- | --- | --- | --- | --- |
| *B. shanianum*-1 | Jiajin Mountain, Yaan, Sichuan | 30°52′15″ | 102°41′00″ | 4200 | Chaozhi 1780503 | MW135451 |
| *B. shanianum*-2 | Baima Snow Mountain, Deqin, Yunnan | 28°05′14″ | 99°05′14″ | 4259 | Chaozhi 1782002 | MW135452 |
| *B. yunnanense*-1 | Lucheng, Kangding, Sichuan | 29°56′13″ | 101°57′47″ | 3120 | Chaozhi 1682301 | MW135450 |
| *B. yunnanense*-2 | Yulong Snow Mountain, Lijiang, Yunnan | 27°10′03″ | 100°14′53″ | 3460 | Chaozhi 1782303 | MW135453 |
| *B. kweichowense* | Fanjing Mountain, Jiangzhong, Guizhou | 27°54′36″ | 108°41′30″ | 2258 | Chaozhi 1882001 | MW135454 |
| *B. rockii*-1 | Yulong Snow Mountain, Lijiang, Yunnan | 27°01′26″ | 100°15′40″ | 2699 | Chaozhi 1782301 | MW135455 |
| *B. rockii*-2 | Dongba canyon, Lijiang, Yunnan | 27°00′30″ | 100°15′43″ | 2672 | TEW008 | MW135456 |

**Table S4** Quantity and quality of the sequencing data and coverage depth of the assembled genomes.

| Sample name | NCBI accession | Data in GB | Whole genome sequencing  reads | Phred score | Chloroplast genome  reads | Coverage mean | Coverage max |
| --- | --- | --- | --- | --- | --- | --- | --- |
| *B. shanianum*-1 | MW135451 | 3 | 24,490,872 | 3,673,630,800 | 150 | 98.22 | 65.735 |
| *B. shanianum*-2 | MW135452 | 3 | 25,384,328 | 3,807,649,200 | 150 | 98.41 | 58.2925 |
| *B. yunnanense*-1 | MW135450 | 3 | 27,759,316 | 4,163,897,400 | 150 | 92.93 | 130.058 |
| *B. yunnanense*-2 | MW135453 | 3 | 25,079,668 | 3,761,950,200 | 150 | 98.13 | 80.3489 |
| *B. kweichowense* | MW135454 | 3 | 22,693,280 | 3,398,323,364 | 150 | 97.20 | 132.28 |
| *B. rockii*-1 | MW135455 | 3 | 23,870,012 | 3,580,501,800 | 150 | 96.89 | 132.231 |
| *B. rockii*-2 | MW135456 | 3 | 23,409,434 | 3,511,415,100 | 150 | 96.20 | 84.6189 |

**Table S5** Taxa included in the molecular analyses, with GenBank accession numbers.

| **Taxon** | **Author(s)** | **GenBank accession** | **Genome size** |
| --- | --- | --- | --- |
| *B. boissieuanum* | Li and Wu, 2017 | NC_036017 | 156108 |
| *B. candollei* | Zhang and Zhao, 2020 | MT261183 | 155541 |
| *B. chinense* | Li et al. 2020 | MN893666 | 155869 |
| *B. chinense* | Zhang et al. 2019 | NC_046774 | 155545 |
| *B. commelynoideum* | Li et al. 2020 | MT162552 | 155629 |
| *B. densiflorum* | Zhang and Zhao, 2020 | MT261184 | 155787 |
| *B. dracaenoides* | Zhao et al. 2020 | MT387201 | 155140 |
| *B. falcatum* | Shin et al. 2015 | NC_027834 | 155989 |
| *B. latissimum* | Kwak 2017 | NC_033346 | 155621 |
| *B. longiradiatum* | Zhang and Zhao, 2020 | MT261186 | 155865 |
| *B. marginatum* | Zhang and Zhao, 2020 | MT261187 | 155610 |
| *B. marginatum* | Yang 2020 | MN968501 | 154991 |
| *B. pusillum* | Zhang 2020 | MT261188 | 155224 |
| *B. scorzonerifolium* | Zhang and Zhao, 2020 | MT239475 | 155824 |
| *B. sibiricum* | Zhang and Zhao, 2020 | MT261190 | 155785 |
| *B. tenue* | Zhang and Zhao, 2020 | MT261191 | 155822 |
| *B. thianschanicum* | Zhang and Zhao, 2020 | MT261192 | 155723 |
| *B. triradiatum* | Zhang 2020 | MT261193 | 155796 |
| *B. yinchowense* | Zhang 2020 | MT261194 | 155458 |
| *Chamaesium paradoxum* | Zheng 2019 | MK780227 | 144558 |
| *Chamaesium spatuliferum* | Zheng 2019 | MN119371 | 153770 |
